# Acute Myocardial Injury and Rhabdomyolysis in COVID-19 Patients: Incidence and Mortality

**DOI:** 10.7759/cureus.18899

**Published:** 2021-10-19

**Authors:** Liaquat Ali, Imran Mohammed, Imran Janjua, Muhammad Naeem, Gholam Adeli, Osama Elalamy, Mohammad Alhatou, Naveed Akhtar, Beatriz Canibano, Ambreen Iqrar

**Affiliations:** 1 Neurology, Hamad General Hospital, Doha, QAT; 2 Neurology, Weill Cornell Medicine, Doha, QAT; 3 Internal Medicine, Hamad General Hospital, Doha, QAT

**Keywords:** myocardial ischemia type 2(mi type 2), myocardial injury, rhabdomyolysis, creatine kinase (ck), angiotensin converting enzyme 2 (ace2), coronavirus disease 2019 (covid-19), severe acute respiratory syndrome coronavirus-2 (sars-cov-2)

## Abstract

Background

Myocardial injury has been defined as an elevated troponin level. The frequency of acute myocardial injury of hospitalized coronavirus disease 2019 (COVID-19) patients ranges from 7% to 36%. COVID-19 patients with cardiovascular disease (CVD) have a four-fold higher risk of mortality (odds ratio, 4.33; CI 95%, 3.16-5.94). In COVID-19 hospitalized patients’ study showed mortality rate was 18.5%. Rhabdomyolysis is considered as muscle necrosis and the release of intracellular muscles elements and enzymes into blood. In one of retrospective cohort study of COVID-19 hospitalized patients, incidence of rhabdomyolysis was 16.7%.

Materials and methods

This retrospective observational study consisted of 413 COVID-19 hospitalized patients. Patients with rhabdomyolysis was defined as creatine kinase level greater than 1,000 U/L and acute myocardial injury was defined as serum high-sensitivity troponin-T for males greater than 30 ng/l and for female greater than 20 ng/l. The primary outcome was in-hospital mortality of COVID-19 patients with acute myocardial injury and rhabdomyolysis.

Results

The incidence of acute myocardial injury and rhabdomyolysis in hospitalized COVID-19 patients was 23.9% (99) and 15.7% (65), respectively. The mortality rate of in hospitalized COVID-19 patients who developed acute myocardial injury (28.3%) was significantly higher in comparison to patients who developed rhabdomyolysis (13.8%).

Discussion

The binding of SARS-CoV-2 virus to the angiotensin-converting enzyme 2 (ACE2) is a critical step in the pathophysiology in patients with COVID-19. There may be diverse direct and indirect mechanisms of acute myocardial injury in COVID-19 including ischemic injury, hypoxic injury (MI type 2), direct viral myocarditis, stress cardiomyopathy and systemic cytokine storm. Musculoskeletal injury may be caused by direct viral myositis or indirectly by host immune hyperinflammatory cytokine storm response that leads to skeletal muscle fiber proteolysis and fibrosis.

Conclusions

Acute myocardial injury and rhabdomyolysis were underreported in COVID-19 patients. The incidence and mortality of acute myocardial injury are higher than that of rhabdomyolysis in COVID-19 hospitalized patients. The outcome was worse in COVID-19 patients with severe acute myocardial injury. Patients with acute myocardial injury and rhabdomyolysis may get benefits from rehabilitation programs.

## Introduction

A novel human RNA coronavirus named as severe acute respiratory syndrome coronavirus-2 (SARS-CoV-2) was the cause of a cluster of pneumonia cases in Wuhan, China, in late 2019 and which was later called COVID-19 (coronavirus disease 2019) [1].

COVID-19 patients have cardiac manifestations including acute myocardial injury, heart failure, cardiogenic shock and arrhythmia. Acute myocardial injury is defined as an elevated troponin level and all conditions causing cardiomyocyte death [2]. The frequency of acute myocardial injury of COVID-19 hospitalized patients is variable from 7 to 36% due to the severity of COVID-19, use of troponin assays, 99^th^ percentile threshold and sampling times [3,4]. COVID-19 patients with cardiovascular disease (CVD) have a four-fold higher risk of mortality (odds ratio, 4.33; CI 95%, 3.16 to 5.94) [5]. In a study of COVID-19 hospitalized patients from New York, USA, the mortality rate was 18.5% [3]. COVID-19 may directly or indirectly affect the cardiovascular system causing acute coronary system (ACS), myocarditis and electrical heart disease.

Rhabdomyolysis is considered muscle necrosis and the release of intracellular muscles elements and enzymes into blood. Rhabdomyolysis is defined as creatine kinase (CK) level more than five times of the upper limit of normal (>1000 IU/L) [6]. In rhabdomyolysis, patients may present with muscles pain or weakness, red to brown color of urine (due to myoglobinuria) and markedly elevated creatine kinase (CK) and myoglobin levels. The other manifestations included electrolytes abnormalities ( such as hyperkalemia, hyperphosphatemia, hyperuricemia, hypocalcemia and metabolic acidosis) and later complications included acute kidney injury (AKI), compartment syndrome and rarely disseminated intravascular coagulation (DIC). Trauma, immobilization, sepsis, exertional heat stroke, generalized tonic colonic seizures, vascular and cardiac surgeries are the most common conditions associated with rhabdomyolysis. Rhabdomyolysis rarely occurs in association with an inflammatory myopathy. Rhabdomyolysis associated with COVID-19 has been reported in a few and some retrospective studies. In one of retrospective cohort study of 140 patients, the incidence of rhabdomyolysis in hospitalized COVID-19 patients was 16.7%. The mortality rate was 47.1% in COVID-19 with rhabdomyolysis as in comparison to 26.4% in COVID-19 without rhabdomyolysis [7]. The objective of this study was to determine the incidence of acute myocardial injury and rhabdomyolysis in patients with COVID-19, its outcome and clinical implications.

## Materials and methods

This was a retrospective observational study at Hamad Medical Corporation (HMC), Doha, Qatar. They were allocated tertiary care multidisciplinary hospitals by the government to manage patients with COVID-19 infection. We obtained data of 413 with COVID-19 from 01 January 2020 to 31 December 2020. Patients were enrolled from hospital electronic medical records (EMR) by applying search criteria. We collected information of patients demographics (age, gender, BMI, nationality), presenting symptoms, comorbidities ( like hypertension, diabetes, obesity, smoking, ischemic heart disease, stroke and chronic kidney disease etc), inpatients laboratory values and electronic discharge summaries. The study was approved by the institutional review committee (MRC-01-20-523) at Hamad Medical Corporation (HMC), Doha, Qatar and patients informed consent were waived due to retrospective study and urgently required new data collection of COVID-19 pandemic, results for improves clinical practice. Inclusion criteria were adult age greater than 18 years and COVID-19 confirmation, which was defined based on the positive result of real-time reverse transcription-polymerase chain reaction (RT-PCR) on both nasopharyngeal and oropharyngeal swab specimens. Rhabdomyolysis was defined as when serum creatine kinase (CK) level greater than 1000 U/L and skeletal muscles injury was defined as when serum creatine kinase level greater than 300 U/L or serum myoglobin level greater than 72 ng/ml with or without myalgia, muscles weakness and cola-colored urine. Acute myocardial injury is defined as peak serum high-sensitivity troponin-T greater than 30 ng/l for males, and greater than 70 ng/l for females during hospitalization. We excluded patients who had evidence of secondary rhabdomyolysis due to traumatic or crush injury, heat-related stroke, Ischemic, exertional skeletal muscles injury, generalized tonic colonic seizures, alcohol abuse, certain medicines and toxic substances that were known to cause acquired myopathies and known inherited myopathies prior to admission. Primary outcome was in-hospital mortality. Routine laboratory testing (Complete blood count(CBC), complete metabolic panel (CMP), prothrombin time (PT)/activated partial prothrombin time (APPT)/ international normalized ratio (INR), urea, creatinine, electrolytes, liver function test (LFT), C reactive protein (CRP), Ferritin, lactate dehydrogenase (LDH), D-dimer, interleukine-6 (IL-6), lactic acid, creatine kinase (CK), myoglobin, troponin etc.), radiologic examination included chest x- rays and chest CT that was performed according to standard clinical practice and CDC Qatar guidelines. The primary objective was to determine the incidence of acute myocardial injury and rhabdomyolysis in COVID-19 hospitalized patients and the secondary objective was to determine the mortality in rhabdomyolysis and acute myocardial injury COVID-19 patients. The data were summarized by descriptive statistics; results were reported as mean, median, interquartile ranges (IQR) and standard deviations as appropriate. Categorical variables were expressed as counts and percentages. Continuous variables were compared by using unpaired Wilcox rank-sum test. Proportions for categorical variables were compared by using X^2^ test. The significant threshold was set at P <0.05. All statistical analyses were done by using the statistical package SPSS 24.0 (SPSS Inc. Chicago, IL). 

## Results

Four hundred and thirteen (413) hospitalized patients with COVID-19 were recruited in this study. The patients demographics are shown in Table 1 and the clinical characteristics are shown in Table 2.

**Table 1:** Demographics (age, gender, nationality). The median age was 52 years and the majority were young males (<54 years) and of Indian nationality.

Age (years)	Frequency(n)	Percentage (%)
22 to 54 (young)	234	56.7%
55-74 (middle age)	156	37.8%
75-84 (old age)	21	5.1%
>85 (older age)	2	0.5%
Gender		
Male	389	94%
Female	23	6.0
Nationality		
Indian	84	20.3%
Bangladesh	52	12.5
Qatari	42	10.1
Nepalese	38	9.2
Filipino	36	8.7
Pakistani	36	8.7
Egyptian	29	7.0

**Table 2:** Clinical manifestations. Clinical manifestations in COVID-19 included fever, cough, SOB, myalgia, headache, chest pain in descending order.

Symptoms	Frequency (n)	Percentage %
Fever	321	77.5%
Cough	279	67.4
Shortness of breath (with hypoxia and required oxygen)	243	58.7
Myalgia	116	28%
Headache	43	10.4
Chest pain	41	9.9
Vertigo	24	5.8
Muscle tenderness and weakness	16	3.9
Anosmia	12	2.9
Dysuria	6	1.4
Cola-color urine	1	0.2

The median age was 52 years (range 22 to 86), the majority were male (94%) and 57% were less than 55 years. The most common comorbid illnesses were hypertension (47.6%), diabetes mellitus (46.9%), obesity (21%), chronic kidney disease (10%), ischemic heart disease (9.7%) and smoker (6.8%) as shown in Table 3.

**Table 3:** Common comorbid illnesses and risk factors for COVID-19. The most common medical comorbid conditions and risk factors for COVID-19 were included; hypertension, diabetes mellitus (DM), obesity, chronic kidney disease, ischemic heart disease and smoker in descending order.

Underlying risk factors	Frequency (n)	Percentage (%)
Hypertension	197	47.6 %
Diabetes	194	46.9%
Obesity	88	21%
Newly diagnosed DM (on admission HbA1c >6.5%)	42	10%
Chronic kidney disease	43	10%
Ischemic heart disease	40	9.7%
Smoker	28	6.8%
Asthma	15	3.6%
Malignancy	12	2.91%

**Figure 1:**
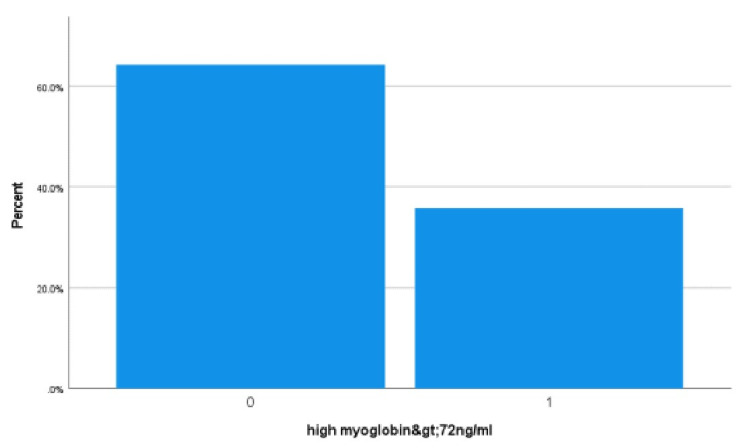
Skeletal muscles injury (myoglobin). In COVID-19 hospitalized patients, there were 35.7% (148) high serum myoglobin (>72 ng/ml) as shown in the bar chart (1 represents greater than 72 ng/ml myoglobin level, zero represents less than 72 ng/ml).

Among all COVID-19 patients, other multiorgan dysfunction included; 37% (153) were cytokine storm, 36.7% (152) were acute kidney injury (AKI), 63.07% (41) were AKI in patients with rhabdomyolysis, 35.7% (148) were skeletal muscle injury (as shown in bar chart Figure 1), 27.5% (114) were acute liver injury, 23.9% (99) were acute myocardial injury, 15.7% (65) were rhabdomyolysis and 11.4% (47) were acute heart failure as shown in Table 4. 45% (187) COVID-19 patients were admitted in MICU, 29% (120) were mechanically ventilated and 16% (67) were managed with noninvasive ventilation (NIV). 8.5% (35) MICU patients died with COVID-19 complications and multiorgan failure. The mortality rate of COVID-19 patients with acute myocardial injury, cytokine storm syndrome and rhabdomyolysis were 28.3% (28), 19.6% (30) and 13.8% (9) in descending order. There were significantly high inflammatory markers (ferritin, CRP, D-dimer and IL-6) in both rhabdomyolysis and acute myocardial injury. The most common ECG changes (23.2%) were sinus tachycardia (5.8%), T waves abnormalities (5.6%), atrial fibrillation(3.6%), asystole (3.6%) in descending order as shown in Table 5.

**Table 4:** Laboratory and echocardiographic findings. Laboratory features associated with severe COVID-19 infection included 71% were high ferritin level, 66.2% were high LDH, 61% were high CRP level, 53.9% were high D-dimer, 39.4% were high CK level, 35.7% were high myoglobin level, 22.2% were high procalcitonin, 15.7% were rhabdomyolysis and 6.3% were high lactic acid. 7.7% hospitalized patients were performed Echocardiography showed 3.4% were mild low EF, 1.7% were severe low EF and 0.5% were moderate low EF.

Laboratory features	Frequency (n)	Percentage (%)
Serum ferritin >500mcg/L	295	71%
Ferritin >500mcg/L in rhabdomyolysis (65)	58	89.2%
Ferritin >500mcg/L in acute myocardial injury (99)	86	86.8%
C-reactive protein levels (>100mg/l)	253	61%
C-reactive protein levels (>100mg/l) in rhabdomyolysis (65)	57	87.8%
C-reactive protein levels (>100mg/l) in acute myocardial injury (99)	86	86.8%
D-Dimer >1000 ng/ml	223	53.9%
D-Dimer >1000 ng/ml in rhabdomyolysis (65)	51	78.5%
D-Dimer >1000 ng/ml in acute myocardial injury (99)	74	74.7%
IL6>70 pg/ml	153	37%
IL6>70 pg/ml in Rhabdomyolysis (65)	42	64.6%
IL6>70 pg/ml in acute myocardial injury (99)	67	67.7%
Acute myocardial injury in patient high inflammatory marker (103) (ferritin, CRP, D dimer, IL-6)	53	51.45%
Rhabdomyolysis in patient high inflammatory markers (103) (ferritin, CRP, D dimer, IL-6)	29	28.15%
LDH >245 units/L	274	66.2%
Serum CK>300u/L	163	39.4%
Serum myoglobin >72 ng/dl	148	35.7%
Acute kidney injury (AKI) (urea>8mmol/L, creatinine>106ml/min)	152	36.7%
Acute kidney injury in rhabdomyolysis (65)	41	63.07%
ALT or AST >200 U/L (Acute liver injury)	114	27.5%
Troponin-T hs (male>30, female>20ng/l) Acute myocardial injury	99	23.9%
Procalcitonin (> 2ng/ml)	90	22.2
Serum CK>1000 U/L (Rhabdomyolysis)	65	15.7%
Pro-BNP (>1800pg/ml) AHF	47	11.4
Lactic acid >4mml/l	26	6.3
Echocardiography		
Mild low ejection fraction (51-41%)	32	7.7
Moderate low ejection fraction (40-30%)	11	2.7
Severe low ejection fraction (<30%)	7	1.7

**Table 5:** ECG changes (413 patients). The most common ECG changes (23.2%) were sinus tachycardia (5.8%), T waves abnormalities (5.6%), atrial fibrillation(3.6%), asystole (3.6%) in descending order.

ECG	Frequency(n)	Percentage (%)
ECG changes (413)	96	23.2%
Sinus Tachycardia	24	5.8%
T waves changes	23	5.6%
Asystole	15	3.6%
Atrial Fibrillation	15	3.6%
AV block abnormalities	10	2.4%
Sinus bradycardia	9	2.2%
ST elevation changes	6	1.4%
Prolonged QTc interval	6	1.4%
Pulseless electrical activity (PEA)	5	1.2%
Ventricular tachycardia (VT)	2	0.5%
Ventricular Fibrillation (VF)	2	0.5%

During the study period of 413 enrolled patients, 89.6% (370) were discharged home and 8.5% (35) died in hospital, and 1.9% (8) were shifted to a rehabilitation center for physical therapy. Among all COVID-19 hospitalized patients with high inflammatory markers (103), 51.45% (53) were acute myocardial injury and 28.15% (29) were rhabdomyolysis as shown in Table 4. There was a significantly higher acute myocardial injury in comparison with rhabdomyolysis in patients with high inflammatory markers (51.45% Vs 28.15%). Among all COVID-19 hospitalized patients, 10.6% (44) were both acute myocardial injury and heart failure, 6.8% (28) were both acute myocardial injury and rhabdomyolysis. The incidence of acute myocardial injury was 23.9% (99) and the incidence of rhabdomyolysis was 15.7% (65) in all COVID-19 hospitalized patients. The mortality rate of COVID-19 hospitalized patients who developed acute myocardial injury was significantly higher in comparison with patients who developed rhabdomyolysis (28.3% vs. 13.8%).

## Discussion

The purpose of this study was to investigate the incidence and mortality of acute myocardial injury and rhabdomyolysis in COVID-19 hospitalized patients in designated COVID-19 treatment centers in Qatar. The most important step in the pathogenesis of COVID-19 is the binding of SARS-CoV-2 to angiotensin-converting enzyme 2 (ACE 2) receptors [8]. ACE-2 has a wide distribution in multiple organs including skeletal and cardiac muscles [9]. Cytokine storm is defined as an activation cascade of auto-amplifying cytokine production due to the unregulated host innate and adaptive immune response activated by SARS-CoV-2 infection. There is increasing evidence that cytokines such as IL-6, IL-1 beta, IFN-gamma and TNF-alpha play important role in pathogenesis of cytokine storms in COVID-19. Patients may acutely deteriorate in cytokine storms and develop acute respiratory distress syndrome (ARDS) and other multiorgan failure (MOF) [10-12]. 

SARS-CoV-2 related viral myositis may be attributable to direct myocyte invasion or induction of autoimmunity. In one of the autopsy series of patients who had died with COVID-19, concluded that most individuals with COVID-19 showed signs of myositis ranging from mild to severe and associated with duration of illness. Inflammation of skeletal muscles was more distinct than cardiac inflammation. Detection of viral load was low or negative in most skeletal and cardiac muscles and probably attributable to circulating viral RNA rather than genuine infection of myocytes. This suggests that SARS-CoV-2 may be associated with a post-infectious, immune-mediated myopathy [13]. There may be various direct and indirect mechanisms of acute myocardial injury in COVID-19 including ischemic injury, hypoxic injury (MI type 2), direct viral myocarditis, stress cardiomyopathy and systemic cytokine storm.

There may be various direct and indirect mechanisms of acute myocardial injury in COVID-19 including ischemic injury caused by cardiac microvascular damage or endotheliitis or epicardial coronary artery disease as thrombotic and plaque rupture or thrombosis due to hypercoagulability, hypoxic injury (supply-demand mismatch as myocardial ischemia type 2), direct viral myocarditis, stress cardiomyopathy and systemic cytokine storm [14,15]. The role of ACE-2 receptor-related signaling pathways dysfunction in COVID-19-related cardiac injury is unknown.

The exact mechanism of skeletal muscle damage with SARS-CoV-2 viral infections is not fully understood. It is unclear if SARS-CoV-2 infects muscles directly [7]. Musculoskeletal injury may be caused by indirect host cytokine storm immune response, leading to skeletal muscle fiber proteolysis and fibrosis [16]. Among all COVID-19 hospitalized patients, 10.6% (44) were both acute myocardial injury and heart failure, 6.8% (28) were both acute myocardial injury and rhabdomyolysis, 36.7% (152) were acute kidney injury (AKI) and 63.07% (41) were AKI in rhabdomyolysis patients. There was a significantly higher acute myocardial injury in comparison with rhabdomyolysis in patients with high inflammatory markers (51.45% vs. 28.15%). In this study, the incidence of acute myocardial injury was 23.9% and the incidence of rhabdomyolysis in COVID-19 patients were 15.7%. The mortality rate of acute myocardial injury (28.3%) was significantly higher in comparison with rhabdomyolysis (13.8%) in COVID-19 patients.

## Conclusions

Acute myocardial injury and rhabdomyolysis are important complications that are underreported among COVID-19 hospitalized patients. Patients with COVID-19 may present with a broad spectrum of cardiac manifestations as no clinical evidence of heart disease, asymptomatic heart disease (have cardiac test abnormalities) and symptomatic heart disease. The common neuromuscular manifestation of COVID-19 patients are myalgias, skeletal muscles injury and rhabdomyolysis. There was a significantly higher acute myocardial injury in comparison with rhabdomyolysis among COVID-19 patients with high inflammatory markers. The incidence and mortality of acute myocardial injury in comparison with rhabdomyolysis were significantly higher among COVID-19 patients. In future, more prospective studies will be required with a focus on short- and long-term outcomes of COVID-19 patients and to get the benefit of using rehabilitation programs.
